# How and why to study autophagy in *Drosophila*: It’s more than just a garbage chute

**DOI:** 10.1016/j.ymeth.2014.11.016

**Published:** 2015-03-15

**Authors:** Péter Nagy, Ágnes Varga, Attila L. Kovács, Szabolcs Takáts, Gábor Juhász

**Affiliations:** Department of Anatomy, Cell and Developmental Biology, Eötvös Loránd University, Pázmány s. 1/C. 6.520, Budapest H-1117, Hungary

**Keywords:** Atg8, Autophagy, Autophagosome, *Drosophila*, Flux, p62/Ref(2)P

## Abstract

During the catabolic process of autophagy, cytoplasmic material is transported to the lysosome for degradation and recycling. This way, autophagy contributes to the homeodynamic turnover of proteins, lipids, nucleic acids, glycogen, and even whole organelles. Autophagic activity is increased by adverse conditions such as nutrient limitation, growth factor withdrawal and oxidative stress, and it generally protects cells and organisms to promote their survival. Misregulation of autophagy is likely involved in numerous human pathologies including aging, cancer, infections and neurodegeneration, so its biomedical relevance explains the still growing interest in this field. Here we discuss the different microscopy-based, biochemical and genetic methods currently available to study autophagy in various tissues of the popular model *Drosophila*. We show examples for results obtained in different assays, explain how to interpret these with regard to autophagic activity, and how to find out which step of autophagy a given gene product is involved in.

## Graphical abstract

**Figure:**
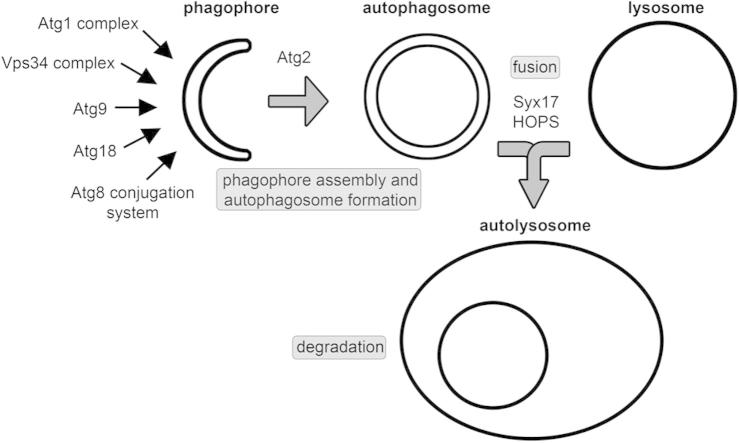

## 1 Introduction

**Fig. 1:**
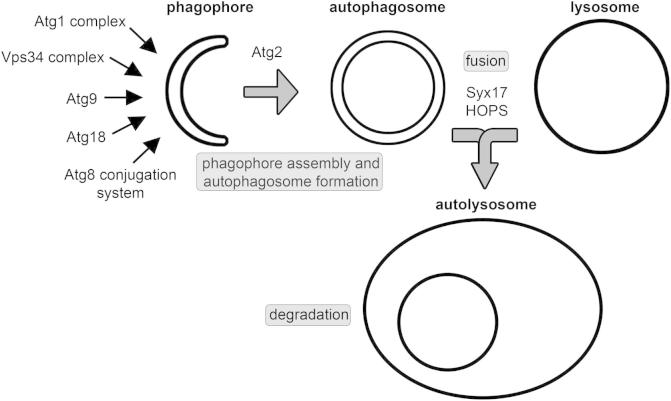
The process and molecular mechanisms of autophagy. The sequential and coordinated action of Atg protein complexes mediates the formation of phagophores and double-membrane autophagosomes. Fusion of autophagosomes with lysosomes (or with late endosomes) requires a Syntaxin 17-containing SNARE complex and the HOPS tethering complex. Autophagic cargo is degraded in autolysosomes, which is followed by recycling of degradation products in synthetic and energy producing pathways.

Eukaryotic cells can deliver portions of their own cytoplasm (including organelles and protein aggregates) for lysosomal degradation in several ways [1]. Macroautophagy (hereafter simply referred to as autophagy) is the best studied and probably most abundant of these routes. Three main steps can be distinguished during this vesicular transport process (Fig. 1). First, a phagophore cistern (also known as the isolation membrane) forms and engulfs cytoplasmic cargo into a double-membrane vesicle called autophagosome. Second, the autophagosome fuses with a lysosome (or late endosome) to give rise to an autolysosome (or amphisome). Third, contents delivered for degradation are broken down by acidic hydrolases within the autolysosome, and the resulting monomers are recycled for biosynthesis and energy production.

The ongoing revolution of this research field started after the discovery of yeast Atg (autophagy-related) genes in the mid-1990’s [2,3]. Most of these genes’ products turned out to be evolutionarily conserved, and form functional complexes to facilitate autophagosome biogenesis [1,2]. As a result, it is now possible to follow the progression of autophagy using transgenic, tagged Atg reporter lines or specific antibodies. Moreover, the role of autophagy can be analyzed by looking at Atg loss-of-function cells/animals using mutants or in RNAi experiments.

As our autophagy-related knowledge and tools have evolved over the past decade, so have the standards of the field. It was appropriate to publish papers on the regulation of autophagy using only electron microscopy before the molecular era, or a vital staining for acidic organelles 10 years ago [4–6]. Without questioning the value of many important papers published earlier, the conclusions of which are still valid, the addition of multiple different tests by the new methods gives more insight and more reliable results. New approaches include assays that also account for the dynamic nature of autophagy, in addition to taking snapshots of the number and type of autophagic structures.

Reviews describing various tests for autophagy in *Drosophila* have previously been published, and the most notable among them is the 2012 guidelines paper aimed at providing a comprehensive overview of all the assays that can be used across species and representing a consensus view of the entire research community [7–9]. In line with the standards set by these publications, here we describe an updated collection of currently available tests that, when used in combination, allow the assessment of autophagic activity in various *Drosophila* tissues.

Autophagy has been studied in several experimental settings in the fruit fly, including starvation-induced and developmental autophagy in the larval fat body, a tissue similar to our liver and fat [6,10]. In the first case, feeding larvae are usually transferred from a rich culture medium to 20% sucrose. After floating on top of this solution and starving for 3–4 h, the fat body is dissected and processed for analysis [6]. In the case of developmental autophagy, third (last) instar larvae are collected in the so-called wandering stage: when animals search for a dry place to pupariate. This change in behavior is triggered by a small peak of the molting hormone ecdysone, which also induces autophagy [10]. Of course, polyploid tissues other than the fat body (such as the midgut) also respond to starvation or ecdysone [10]. In contrast, diploid imaginal cells usually show much lower levels of autophagy during starvation, which is likely due to sustained local paracrine growth signaling and to the autophagic remobilization of nutrients stored in polyploid cells [11]. During the five-day non-feeding period of metamorphosis, diploid cells proliferate and differentiate to form the tissues and organs of the adult fly. This again is likely to rely on nutrients liberated from polyploid cells that undergo massive autophagy and are ultimately eliminated [5]. Apart from studies carried out in larvae and pupae, autophagy has also been analyzed in embryonic tissues (e.g. amnioserosa) and adult flies (e.g. brain, ovary, testis) [12–19]. Importantly, while the application of the various methods may slightly differ among fly tissues or between flies and other eukaryotes, the interpretation of results from these tests always follows the same logic.

## 2 Transmission electron microscopy and the morphology of autophagy

The process of autophagy was discovered by ultrastructural analysis, based on the presence of still recognizable cytoplasmic structures (such as RER and mitochondria) in various states of degradation within lysosomes [20]. Double-membrane autophagosomes were identified as the transport vesicles delivering self-material into lysosomes, as fusion events were captured between them [20]. Even these days, electron microscopy is the only technique that allows the visualization of autophagic structures at high resolution: for example, distinguishing the two limiting membranes of autophagosomes is still far beyond the resolving power even of superresolution light microscopes.

Unfortunately, classical transmission electron microscopy is less frequently used these days, and the number of institutional core facilities that can process regular ultrathin sections to analyze cells and tissues is decreasing. Ultrastructural analysis requires expertize in evaluation, experienced personnel and a setup that is expensive to establish and maintain. Despite these limitations, we strongly believe that transmission electron microscopy is still very important in autophagy studies, because it greatly helps the proper interpretation of data from biochemical and fluorescent microscopy experiments, and is indispensable in many cases to achieve reliable results [20].

**Fig. 2:**
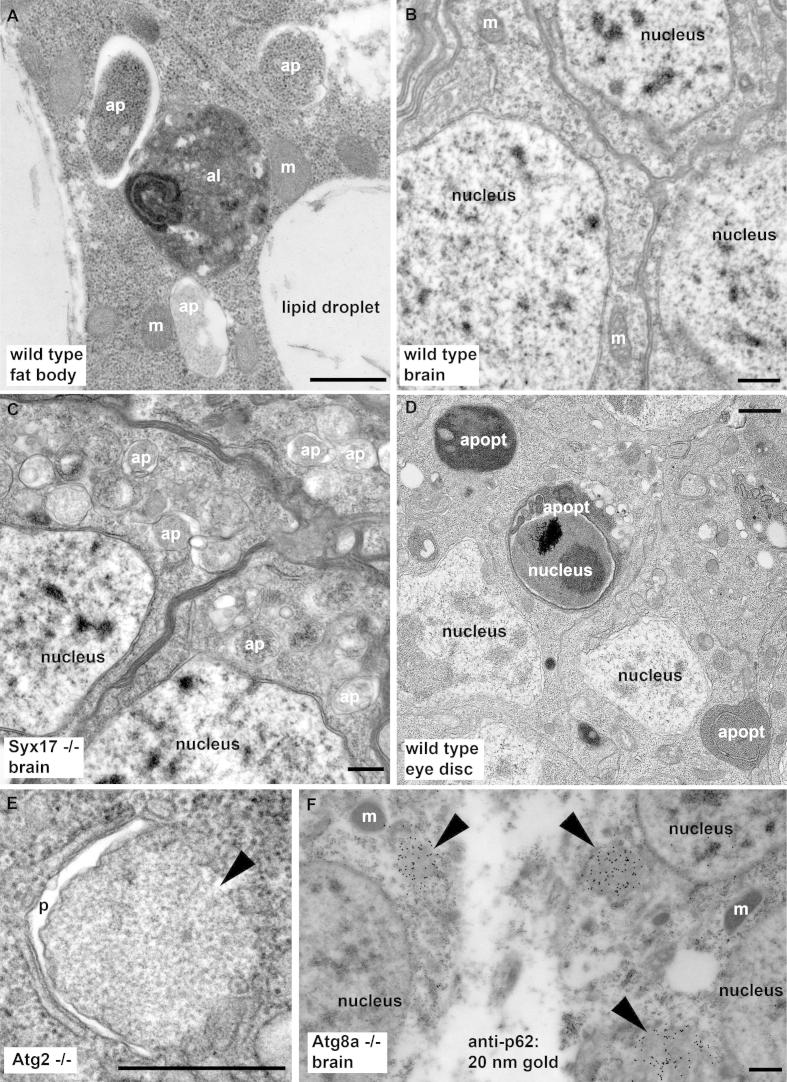
Ultrastructural analysis of autophagy. (A) Transmission electron microscopy image of fat body from a starved larva. Autophagosomes (ap) bordered by a double membrane (with the often seen characteristic cleft between them) contain undegraded cargo, which appears largely similar to the surrounding cytoplasm. In contrast, the content of the single-membrane bound autolysosome (al) is typically dark and condensed due to ongoing breakdown, although remnants of cytoplasmic material can often be recognized. (B) Autophagic structures are practically never seen in ultrastructural images of wild type adult brains. (C) Double-membrane autophagosomes accumulate in large numbers in neurons of Syntaxin 17 mutant adult flies. (D) Apoptotic bodies (apopt) induced by clonal overexpression of *Hid* are engulfed by neighboring eye imaginal disc cells, and contain cytoplasmic remnants of the fragmented dying cell, but these are not of autophagic origin. Their recognition is facilitated by the presence of the condensed nucleus in some of the engulfed cell fragments, and apoptotic bodies may be found outside of healthy cells as well, as illustrated by the one situated between two neighboring cells in the bottom right corner of this panel. Note that apoptotic cells usually appear darker than healthy cells in ultrastructural images even before being engulfed, due to ongoing protein degradation by caspases and acidification of the cytoplasm. (E) Large protein aggregates form in neurons of Atg mutant adult flies. The aggregate (arrowhead) can be easily recognized by its homogenous appearance, as they mostly exclude cytoplasmic structures, for example vesicles and ribosomes. This image shows a single protein aggregate with a rarely seen phagophore (p) attached to its surface in an Atg2 mutant brain. (F) Protein aggregates (arrowheads) accumulating in Atg mutant neurons contain ubiquitinated proteins and their selective autophagic receptor p62 (also known as Ref(2)P in flies). This image shows immunogold labeling (black dots) of p62 in Atg8a mutant neurons. Abbreviations: ap, autophagosome; apopt, apoptotic cell fragment; al, autolysosome; p, phagophore; m, mitochondrion. Bars equal 1 μm in all panels.

Autophagic structures have certain unique features that make it possible to reliably recognize them. Depending on the sample preparation technique and type of fixation used, the closely apposed membranes of both phagophore cisterns and autophagosomes may open up, and thus a characteristic cleft appears between the two membrane sheets (as seen in Fig. 2A,C and E; note that such vesicles are very rarely observed in neurons of wild type adult flies, shown in Fig. 2B). This phenomenon is often seen in samples prepared by chemical fixation of cells and tissues by a glutaraldehyde-containing isoosmotic solution, which is followed by dehydration of fixed samples and embedding into a resin [6,10,13,21–23]. Intracellular structures likely undergo a variable extent of shrinkage during fixation and embedding according to local conditions in the surrounding cytoplasm, which is why this cleft may be seen only in a subset of autophagic structures. The appearance of this cleft is unlikely to be specific for different cell types or organisms, as it has been documented elsewhere as well [9,24]. The membranes of these early autophagic structures are of the thin type, based on which autophagosomes can be distinguished from interdigitations observed between neighboring cells in some tissues, as the membrane of these are of the thick type (plasma membrane). Autophagosomes contain undigested material, the morphology of which appears very similar to the surrounding cytoplasm (Fig. 2A and C). In contrast, the content of autolysosomes is mostly heterogeneous, as it shows morphological signs of ongoing degradation ranging from recognizable to finally unidentifiable cytoplasmic material like mitochondria, RER, ribosomes and so on. The heterogeneous morphology can be the result of multiple subsequent fusion events resulting in a multifocal appearance. Both primary lysosomes and actively digesting autolysosomes and endolysosomes contain acid phosphatase, which can be detected in the electron microscope using a classical, very simple enzymatic reaction [25,26]. In addition, immunogold labeling for lysosomal proteins using antibodies (or tagged reporters and anti-tag antibodies) to cathepsin proteases or lysosomal membrane proteins can also be used to identify lysosomes in ultrastructural studies [26].

The late phase autolysosomes in *Drosophila* often appear as overwhelmingly dark, homogenous bodies where the final stage of digestion takes place. Large spherical autolysosomes with a diameter of up to 10 μm are especially characteristic for developmental autophagy of the fat body, probably because final degradation of sequestered material is delayed to provide a continuous supply of breakdown products during metamorphosis. A word of caution is that it is very important to properly distinguish autolysosomes from engulfed apoptotic bodies that also contain cytoplasmic material, but in this case it originates from the dying cell and is not a result of autophagy. Engulfment can be carried out not only by professional phagocytes but also by neighboring cells such as in the developing eye (Fig. 2D). In this example, large endocytosed fragments of the dying cell reach the lysosome through the heterophagy pathway, and thus show no heterogeneity or multifocality. Moreover, cell fragments may contain the condensed nucleus, are often found outside of engulfing cells as well, and the cytoplasm of apoptotic cells and fragments usually appears dark even before reaching the lysosome, due to ongoing degradation through caspase activity and acidification (Fig. 2D).

In addition to the qualitative analysis of autophagic structures, ultrastructural data can also be quantified. This should be based on measuring the area of autophagic vesicles in the sections compared to total cytoplasmic area around them, and following a random sampling method. Counting only the number of vesicle profiles per cell in sections without measuring their area and that of the containing cytoplasm leads to false results, because smaller organelles appear less frequently in 2-dimensional sections than bigger ones [27].

## 3 Fluorescent microscopy and Atg gene products

Though important and often indispensable, ultrastructural analysis is insufficient to deal with the biological variability and heterogeneity of an organ/tissue when only a few cells may show a given phenotype, or to screen lots of different genotypes. Applying electron microscopy for autophagy research is somewhat like finding a needle in a haystack, so it is generally advisable to have an idea of what we are looking for before starting such scrutiny. This can be achieved by fluorescent microscopy analysis. Certain membrane-permeable vital dyes that accumulate inside acidic vesicles, such as Lysotracker Red and Acridin Orange, have been successfully used for this purpose [28]. While these stains are not specific for autophagy in general, Lysotracker Red has proven to be a widely used and apparently quite reliable test in larval *Drosophila* fat body [6–8,10] and ovary [16,19]. Fat body cells show little to no staining in well-fed larvae, perhaps because their lysosomes are normally very small and/or not acidic. In contrast, Lysotracker-positive autolysosomes suddenly appear in these cells during starvation-induced or developmental autophagy. However, this staining is not so useful in some other tissues: for example, larval nephrocytes (Garland and pericardial cells) are highly endocytic and their lysosomes always stain positive for Lysotracker (and Acridin Orange). Lysosomes can also be labeled using an artificial GFP-Lamp reporter that only contains the lysosome targeting and transmembrane sequences of human Lamp1 (lysosome-associated membrane protein 1) fused to GFP. This marker is transported to lysosomes and is degraded there, and a block of trafficking to lysosomes results in its accumulation [29]. This tool allows the visualization of lysosome numbers and distribution, and it is often useful for determining the identity of autophagosomes versus autolysosomes in colocalization studies with Atg8a. [8]. However, it should not be used as a specific marker for autophagy on its own.

Atg protein-based tests are considered to be more specific than Lysotracker staining, and these can be used in all cell types. A number of transgenic GFP- or mCherry-tagged autophagy reporter lines have become available during the past decade, and samples can also be labeled using indirect immunofluorescence utilizing published antibodies against endogenous proteins. The first specific marker for autophagy was LC3, one of the homologs of yeast Atg8 in mammals, and it is still the most widely used tool in microscopy-based assays [9,30,31]. These small ubiquitin-like proteins associate with autophagosome (and phagophore) membranes through a phosphatidyl-ethanolamine anchor covalently attached to their C-terminal glycine residue, which becomes exposed as a result of proteolytic processing by Atg4. Transgenic *Drosophila* lines expressing GFP-tagged reporters for fly Atg8a, Atg8b and human LC3 proteins were subsequently generated and used for the analysis of starvation-induced and developmental autophagy in the larval fat body [6,10].

Following the distribution of these ubiquitin-like Atg8 family proteins is the most popular approach for microscopy-based analysis of autophagy in various models, partly because they act relatively downstream in the hierarchy of Atg proteins during autophagosome formation (Fig. 1). It is commonly accepted that activation of the Atg1 protein kinase complex initiates phagophore formation, which is accompanied by the action of a Vps34-containing lipid kinase complex that generates phosphatidyl-inositol 3-phosphate (PI3P) on forming phagophores. Phospholipid effectors include Atg18 family proteins, and these further promote the assembly of the phagophore. Recruitment of the only transmembrane protein among Atg gene products, Atg9, is also an early step of autophagy induction, and it likely acts as a vesicular transport pathway essential for membrane delivery to the growing phagophore [32,33]. The activation and trafficking of these upstream factors is followed by the recruitment of lipidated Atg8, which is facilitated by ubiquitination-like protein conjugation systems with Atg7 and Atg3 acting as E1 and E2 enzymes, respectively. Atg8 remains associated with autophagosomes, whereas the E3-like complex of Atg5-Atg12 and Atg16 (with Atg12 being the second ubiquitin-like Atg protein besides Atg8) is only associated with phagophores but not with autophagosomes [1,5]. Interestingly, Atg2 appears to act more downstream than Atg8 in metazoan cells, as its loss results in the more frequent appearance of Atg8-positive phagophore-like membranes than seen in control cells (Fig. 2E) [34–36].

**Fig. 3:**
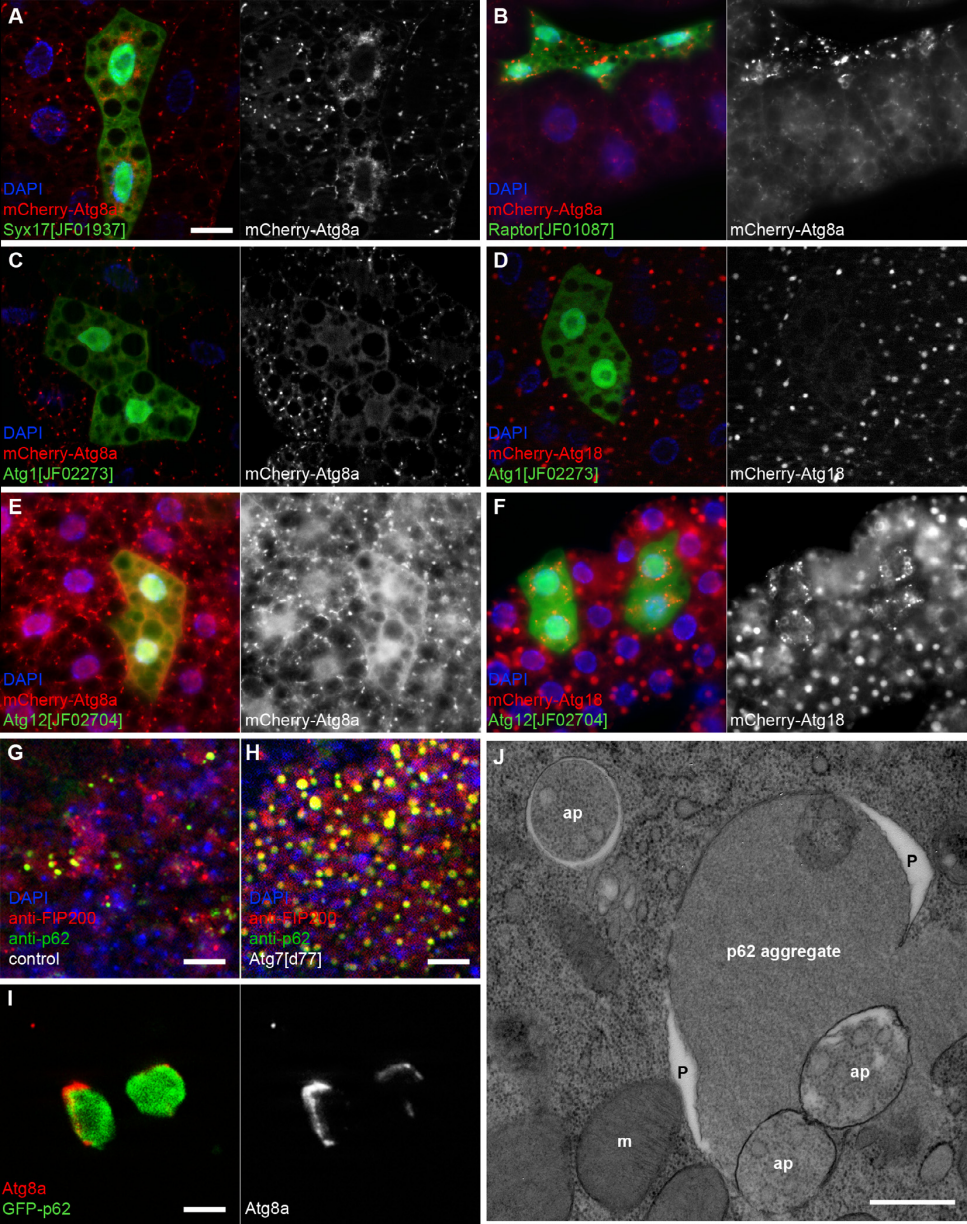
Specific markers of autophagy. (A) The most popular markers of autophagy are fluorescently tagged Atg8a reporters, such as mCherry-Atg8a. Atg8a is delivered to autolysosomes, where mCherry retains its fluorescence and accumulates to high levels. Thus, autolysosomes are labeled bright red in GFP-negative control cells, whereas autophagosomes appear fainter and smaller, as seen in the two GFP-positive cells expressing Syntaxin 17 RNAi, which silences the SNARE required for autophagosome-lysosome fusion. (B) Inhibition of Tor kinase increases autophagic flux, resulting in enhanced delivery of mCherry-Atg8a to lysosomes. Thus, autolysosomes are labeled much brighter with mCherry in GFP-positive cells in which *Raptor* (encoding an essential subunit of Tor kinase complex 1) is knocked down than in surrounding control cells. Note that mCherry signal in control cells looks fainter than in panel A because this image was taken using a much shorter exposure time. (C) and (D) Atg1 RNAi in GFP-positive cells inhibits puncta formation of both mCherry-Atg8a (C) and mCherry-Atg18 (D), as Atg1 acts upstream of Atg8a and Atg18 in the hierarchy of Atg proteins. (E) Knockdown of Atg12 blocks the generation of mCherry-Atg8a dots. (F) Small mCherry-Atg18 dots likely representing stalled phagophore assembly sites (PASs) accumulate in GFP-positive cells undergoing Atg12 RNAi, as Atg18 acts upstream of Atg12. (G) and (H) Protein aggregates containing p62 (green) accumulate in large numbers in the brain of Atg7 mutant flies compared to controls. Note that association of the upstream-acting Atg1 kinase subunit FIP200 (red) with p62 is also increased in Atg7 mutants, suggesting that such aggregates might represent stalled phagophore assembly sites. (I) Phagophores that are positive for endogenous Atg8a associate with large protein aggregates formed upon high-level overexpression of GFP-p62. (J) Ultrastructural analysis demonstrates an enlarged phagophore (P) attached to the surface of a protein aggregate in a fat body cell of a starved larva as in panel I. ap: autophagosome; m: mitochondrion. Panels A–F, I, J show fat body cells of starved L3 stage larvae, and panels G, H show adult brains. Bars equal 20 μm in panels A (for A–F), G, H. Bar in I equals 4 μm, and bar in J represents 1 μm, respectively.

It is worth mentioning that there are two paralogs of yeast Atg8 in *Drosophila*. Atg8a is highly expressed in all tissues, whereas Atg8b only shows strong expression in the testis [37]. Thus, most assays rely on tagged Atg8a. A particularly popular marker for studying autophagy in *Drosophila* is mCherry-Atg8a, because it allows the visualization of all autophagic structures: phagophores, autophagosomes and autolysosomes [38]. This is because Atg8 family proteins are bound to both the inner and outer membranes of autophagosomes, so half of these molecules are delivered to the lysosome in each autophagosomal cycle. Likewise, fluorescently tagged Atg8a also gets into the acidic lysosomal lumen, where GFP is quenched rather quickly but mCherry retains its fluorescence much longer. This is due to their different pKa values [39]. Thus, GFP-Atg8a labels phagophores and autophagosomes more specifically, and only a subset of autolysosomes may be positive for this reporter [6,10,21,30]. In contrast, mCherry-Atg8a accumulates to high levels in autolysosomes, so phagophores and autophagosomes appear fainter (Fig. 3A and B). It is worth noting that the intensity of the mCherry signal may be used to estimate the rate of autophagic protein delivery to lysosomes in these experiments, as a block of autophagosome-lysosome fusion by knocking down the autophagosomal SNARE Syntaxin 17 prevents the formation of highly fluorescent autolysosomes (Fig. 3A) [40], whereas enhanced autophagy due to Tor kinase inhibition results in extremely high levels of mCherry in autolysosomes. As a consequence, these appear much brighter in Tor inactivated cells than the structures seen in surrounding control cells (Fig. 3B). However, sometimes there may be problems with the specificity of overexpressed Atg8 reporters. For example, high-level expression of Atg8a was found to rescue the autophagy-inhibiting phenotype of dominant-negative Atg4 [41]. More importantly, overexpressed Atg8a reporter molecules are captured into the large protein aggregates that form in fat body cells for example during proteasome inhibition [42]. These Atg8a-positive structures may as well be falsely interpreted as large autophagic vesicles, but ultrastructural analysis clearly showed that they are in fact cytosolic protein aggregates. In addition, protein aggregates in proteasome RNAi cells did not stain positive for endogenous Atg8a [42]. Except this unusual situation, indirect immunofluorescence analysis of Atg8a using a specific antibody should give a labeling similar to GFP-Atg8a. One advantage of this technique is that it is based on following the endogenous protein instead of an overexpressed tagged reporter. It is also much faster to carry out, as one does not need to cross the reporter into the genetic background of interest. Several anti-Atg8a antibodies have been published that work well for microscopy in *Drosophila* [16,40,43,44]. Of note, a GFP-Atg8a reporter expressed from the genomic promoter of Atg8a has also been published, which can be used to visualize autophagosomes in the midgut [45]. Unfortunately its expression level is relatively low, and we could not detect it in the larval fat body (our unpublished results). Alternatively, a heat shock-inducible GFP-Atg8a transgene may be used in a sort of pulse-chase experiment to identify autophagosomes and follow their degradation over time [6].

One of the major advantages of the *Drosophila* model is that it is very straightforward to carry out mosaic analysis [7,8]. In this approach, patches of cells are made homozygous mutant, or alternatively, a subset of cells or tissues overexpress either a certain gene to generate gain-of-function phenotypes or a transgenic RNAi construct to silence a gene of interest. Overexpression or RNAi knockdown is usually achieved with the help of a Gal4 transgene that is expressed in a spatiotemporal pattern dictated by the specific promoter used, in combination with a UAS transgene whose expression responds to Gal4. Mutant or Gal4/UAS clones of cells can also be generated randomly with the help of Flp recombinase and its recombination target FRT sites. These cells are usually recognized based on a visible marker such as GFP, or vice versa, by the lack of marker expression (please see Fig. 3A–F for examples). While the detailed discussion of such routinely used genetic manipulations in *Drosophila* is beyond the scope of the present review that focuses on the study of autophagy, it is important to emphasize some of the advantages of mosaic analysis. First, it allows the study of loss- and gain-of-function conditions that cause early lethality when applied on the organismal level. Second, the cell-autonomous nature of a given phenotype can be demonstrated using this technique, to exclude the possibility of an indirect, systemic effect (such as developmental delay). Third, genetically altered cells are situated next to control cells in the same tissue/animal, which allows the direct comparison of mutant and wild-type phenotypes in the same image. Thus, variability from one animal to the other can be dealt with this way.

It is worth noting that Atg proteins other than Atg8 can also be utilized for the visualization of autophagic structures. Atg5 and Atg16L1 are often used to label phagophores in mammalian cells, because these proteins are known to dissociate from mature autophagosomes [9]. A GFP-Atg5 reporter and anti-Atg5 antibodies have been used for this purpose in *Drosophila* as well [10,21,40,46]. Still, it is worth noting that Atg16L1 and Atg5 have been reported to associate with vesicles transporting membrane to growing phagophores and to function in the nucleus, respectively, so these are not exclusively found on phagophores [47,48]. An antibody is also available for the recently identified autophagosomal SNARE Syntaxin 17 [40], but we do not think that this is a specific marker for these vesicles. First, Syntaxin 17 is also found in the ER and potentially in other compartments. Second, this SNARE has been shown to act as a competence factor for autophagosome fusion events, so autophagosomes likely fuse with lysosomes soon after acquiring Syntaxin 17 [40,49].

The mCherry-tagged version of the phospholipid effector Atg18 shows a distribution that is usually indistinguishable from that of mCherry-Atg8a in *Drosophila*. The silencing of Atg1 blocks puncta formation of both reporters (Fig. 3C and D). However, the difference between these markers becomes obvious when one looks at cells lacking genes required for Atg8 lipidation. RNAi-mediated knockdown of Atg12 prevents the formation of Atg8a puncta as expected, whereas mCherry-Atg18 forms small dots that likely represent phagophore assembly sites (Fig. 3E and F). Although experiments of this kind to analyze factors required for the recruitment of certain proteins to autophagic structures are different from classical epistasis tests, these are widely used to infer the hierarchical relationships of Atg proteins [1,32,43,50]. The results shown in Fig. 3 support the accepted functional order of these gene products, according to which Atg1 acts most upstream, followed by Atg18, then Atg12, and finally Atg8a.

## 4 Selective autophagy

Atg8 family proteins are selectively engulfed into autophagosomes due to being physically attached to the inner membrane through their lipid tail. Atg18 homologs have also been reported to associate with PI3P-containing autophagosomal membranes [51], and thus may be subject to specific autophagic degradation, in line with the distribution of the mCherry-tagged reporter.

Pioneering studies in mice and later in *Drosophila* established that ubiquitinated protein aggregates accumulate in neurons upon loss of core Atg genes, indicating that the proteasome is not the only pathway essential for the degradation of ubiquitinated proteins [13,14,52,53]. A landmark discovery identified the first intracellular receptor for the selective autophagic breakdown of ubiquitinated proteins: the multidomain protein p62 (also called sequestosome-1 in mammals and Ref(2)P in flies) [54]. Interaction of p62 with ubiquitin is mediated by its C-terminal UBA domain, and its N-terminal PB1 domain promotes homooligomerization (that is, aggregate formation). A short peptide found in an unstructured region of p62 can bind to a groove on the surface of Atg8 family proteins, and thus it is usually referred to as a LIR/AIM (LC3-interacting region/Atg8 interacting motif) sequence [54]. The binding of the aggregate to phagophores is greatly strengthened by multiple interactions between p62 molecules exposed on the surface of the protein aggregate and lipidated Atg8 family proteins bound to the phagophore membrane. These interactions might also contribute to curving the phagophore, which is essential for its closure into an autophagosome (see Fig. 2E).

Selective autophagy is also involved in the recognition of other cargoes such as intracellular pathogens and mitochondria, or in the proper trafficking of the vacuolar (lysosomal) hydrolase pro-aminopeptidase I in yeast [9,55]. This latter pathway, usually referred to as Cvt (cytoplasm to vacuole targeting), appears to ensure the specific transport of this pro-enzyme in well-fed, exponentially growing yeast cells. In response to starvation, Cvt is switched to the main (bulk) autophagy pathway, which also delivers pro-aminopeptidase I aggregates to the vacuole in a selective but non-exclusive manner [9]. In this regard, the continuous basal autophagy of ubiquitinated protein aggregates in animal cells may be somewhat similar to the Cvt pathway. Likewise, the degradation of these aggregates during autophagy induction appears to remain specific [35,56].

Selective cargoes such as p62 play a very important role in the study of autophagic activity. Disruption of any step of autophagy prevents the proper degradation of p62, leading to large-scale accumulation of its aggregates [9]. As the buildup of p62 is progressive, changes in its levels are summed over the course of a few days, so the effect of an autophagy defect is more obvious in adult flies than in larvae (Fig. 3G and H) [18,40,41,57,58]. Thus, the accumulation of p62 can be used as a specific readout of basal autophagy defects. Larger aggregates of ubiquitinated proteins and p62 forming in autophagy-deficient cells have a very characteristic ultrastructural appearance, which makes it easy to recognize them even without specific immunogold labeling (Fig. 2E, F and Fig. 3J) [13,35]. Vice versa, decreased p62 levels may indicate even slight increases in autophagy over time (e.g. in a few weeks) [59]. Importantly, the indirect and progressive nature of this assay often gives more obvious results than directly looking at autophagic structures based on an Atg8 reporter or ultrastructure: if autophagy levels are low, a snapshot of the actual number of autophagic structures may be difficult to interpret. An often ignored issue about the evaluation of endogenous p62 levels is that the transcription of this gene also changes in response to certain stimuli (such as starvation), which makes it difficult to rely on it as a single assay [37,60]. This problem may be circumvented if the UAS- GFP-p62 reporter is expressed by a constitutive driver (for example actin-Gal4 or a fat body-specific collagen-Gal4) [38,41]. Importantly, known autophagy mutants may be included as positive controls in anti-p62 or GFP-p62 microscopy or western blot experiments. Unfortunately, the high-level expression of p62 by the UAS/Gal4 system in fat body cells leads to large-scale aggregation and co-aggregation with Atg8a reporters, which may lead to difficulties with interpreting data obtained in microscopy [41].

Interestingly, it has been recently reported that giant phagophores are generated in starved yeast cells overexpressing the already mentioned selective autophagy cargo pro-aminopeptidase I. This technique has been successfully used to map the exact localization of various Atg proteins along the enlarged phagophores that are essentially stuck onto the surface of these giant aggregates, being unable to capture them [61]. We have recently found that elongated, aggregate-associated phagophores are also generated in fat body cells of starved *Drosophila* larvae that highly overexpress the autophagy cargo p62 (Fig. 3I and J), which may also be applied for such high-resolution localization studies in animal cells.

Autophagy had been considered to be a non-selective degradation process for a long time, so characterization of specific autophagic cargoes only started in the last few years. A recently described bioinformatic prediction platform may help finding new LIR-containing binding partners of Atg8, the experimental verification of which may be relatively straightforward [62]. For example, putative cargoes should accumulate upon inhibition of autophagy, and their physical binding to Atg8 should be lost if key residues of the suspected LIR sequence are mutated.

## 5 Western blot

Microscopy-based assays are sometimes the only possible approach to study autophagy, for example when working with mosaic animals. Still, it is usually feasible to collect enough samples from mutant larvae or adults, or in transient overexpression or tissue-specific RNAi experiments for western blot analysis. Western blots complement microscopy-based data very well for the evaluation of autophagy, and these results greatly improve the reliability of a new finding as they are obtained using a completely different experimental approach. It is not ideal to rely only on western blots when assessing autophagy in *Drosophila* though, because systemic non-cell-autonomous effects cannot be excluded this way.

**Fig. 4:**
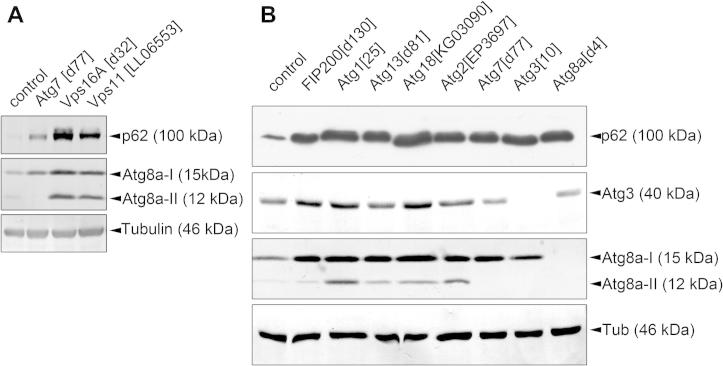
Western blot analysis of autophagy. (A) The autophagic cargo p62 accumulates in starved larvae lacking the core autophagy gene Atg7, and also in animals that are mutant for *Vps16A* or *Vps11,* encoding subunits of the HOPS tethering complex. Autophagosome-associated, lipidated Atg8a-II is missing in Atg7 mutants but accumulates in HOPS loss-of-function (mutant for *Vps16A* or *Vps11*) larvae, as this tethering complex is required for the fusion of autophagosomes with lysosomes (compare Atg8a-II levels to tubulin in controls and mutants, respectively). (B) Various Atg mutants all accumulate p62, whereas the levels of Atg8a-II are increased in starved larvae lacking Atg1, Atg13, Atg18 and Atg2. Atg7 and Atg3 encode the E1 and E2 enzymes required for Atg8a lipidation, respectively, so the generation of Atg8a-II is blocked in these mutants. Both forms of Atg8a are missing in Atg8a mutants, as expected.

The two most commonly used endogenous proteins evaluated in western blots are again Atg8 and p62 in *Drosophila*. The increased amount of p62 may indicate a block of autophagy (Fig. 4A and B) (note that the fly protein is larger than its mammalian homologs and migrates near 100 kDa) [18,40,41,57,58,63]. The level of p62 that can be detected in western blots is strongly influenced by sample preparation. Serial detergent extraction experiments revealed that not all of the p62 pool is recovered in a non-ionic detergent (such as Triton X-100) fraction, since aggregates containing p62 and ubiquitinated proteins that accumulate upon inhibition of autophagy must be solubilized by ionic detergents such as SDS [57]. We routinely boil the samples that we collect (such as fly heads or whole animals) in an SDS-containing Laemmli buffer for 3 min, which is followed by homogenization and another round of boiling to recover as much protein as possible. Even under these harsh extraction conditions, some of the p62 pool likely remains aggregated in autophagy mutant heads, based on p62 signal at a very high molecular weight in western blots [41].

In the case of endogenous Atg8a, the western blot assay is based on the fact that its lipidation increases its speed of migration during polyacrylamide gel electrophoresis. Thus, the non-processed form (often referred to as Atg8a-I) can be easily distinguished from the membrane-associated, faster migrating active form (Atg8a-II). This assay is standard in cultured mammalian cells, and it is becoming more frequently used in flies as well with the availability of multiple specific antibodies [14,16,40,44,63–65]. An increase in the amount of Atg8a-II relative to a loading control (such as tubulin) usually indicates increased autophagosome numbers, as is the case in mutants lacking HOPS complex subunits required for the tethering of autophagosomes with lysosomes (Fig. 4A) [63]. However, the increased levels of Atg8a-II can be either due to increased autophagosome formation or decreased autophagic degradation, or a combination of both (please see the next chapter of this paper for a discussion of autophagic flux) [9]. Another problem is that lipidated Atg8a may show large-scale accumulation in certain Atg mutants (Fig. 4B). While the exact nature of the membranes that likely contain Atg8a-II in these mutants is incompletely understood, similar observations have been reported in *Caenorhabditis elegans* [36]. Thus, some caution needs to be exercised again when interpreting Atg8a immunoblotting, and it is always important to carry out additional tests in order to reliably estimate autophagic degradation.

Others and we have recently reported that the phosphorylation status of *Drosophila* Atg13 depends on Atg1 kinase activity and very well correlates with autophagy status [38,43]. Endogenous Atg13 becomes hyperphosphorylated during autophagy induction, genetic inhibition of the autophagy suppressor Tor, or in response to overexpression of Atg1 [43]. While other kinases likely also regulate Atg13, anti-Atg13 western blots may prove useful for following Atg1 kinase activity in *Drosophila* fat body cells.

## 6 The concept of autophagic flux

The actual number of autophagosomes and autolysosomes depends on both their rate of formation and disappearance. For example, loss of the autophagosomal SNARE Syntaxin 17 results in large-scale accumulation of autophagosomes because this protein is required for autophagosome-lysosome fusion, and it likely does not affect the rate of autophagosome formation [40,66]. In contrast, rapamycin treatment that inactivates Tor, a central kinase promoting cell growth and inhibiting autophagy by direct phosphorylation of Atg1, increases autophagic turnover (flux) and not necessarily the actual number of autophagic vesicles (see Fig. 3B) [67]. Thus, it is very important to carry out proper assays that deal with the dynamic nature of autophagy. One obvious candidate approach is videomicroscopy, but applying this technique is challenging in live animals, especially for longer periods of time or recording simultaneously in two color channels.

The most frequently used tests for autophagic flux are the previously described microscopy and western blot assays for p62, which accumulates to high levels over time if autophagy is disrupted [18,35,40,41,43,57,58,63,64,68,69]. These generally give a quite reliable measure of autophagic degradation rate, provided that the transcription of p62 does not change in the given experimental setting. Mosaic analysis can again provide a reliable, built-in control to account for the starvation-induced transcriptional upregulation of p62 for example [38,40,41,63]. The levels of p62 usually well correlate with that of ubiquitinated proteins, so measuring this latter may be also used to estimate autophagic (and/or proteasomal) flux [35,42,57].

A widely used assay in mammalian cells is to transiently block autolysosomal degradation, by using lysosome inhibitors such as chloroquine or bafilomycin A1 (note that this latter drug may also inhibit autophagosome-lysosome fusion for unknown reasons) [9]. Both microscopy of (GFP-)LC3 or western blot of LC3-II can be evaluated, by comparing dot numbers or band intensity in the presence of the drug to the condition without drug treatment, respectively [9]. This strategy is similar to plugging the sink when the water is running from a tap, which makes it easier to estimate the amount of incoming material. Chloroquine can also be used for this purpose in Atg8a assays in *Drosophila*, although prolonged treatment of animals with this drug may also result in secondary effects including a myopathy-like phenotype [42,44,70]. Another problem with these assays is that the inhibition of lysosomal degradation inactivates Tor kinase, which results in increased autophagosome formation and may complicate the proper interpretation of results [71–73]. The loss of Syntaxin 17 may turn out to be a useful genetic tool to specifically inhibit autophagosome-lysosome fusion without affecting lysosomal function in *Drosophila*, but this needs to be further verified experimentally [66].

**Fig. 5:**
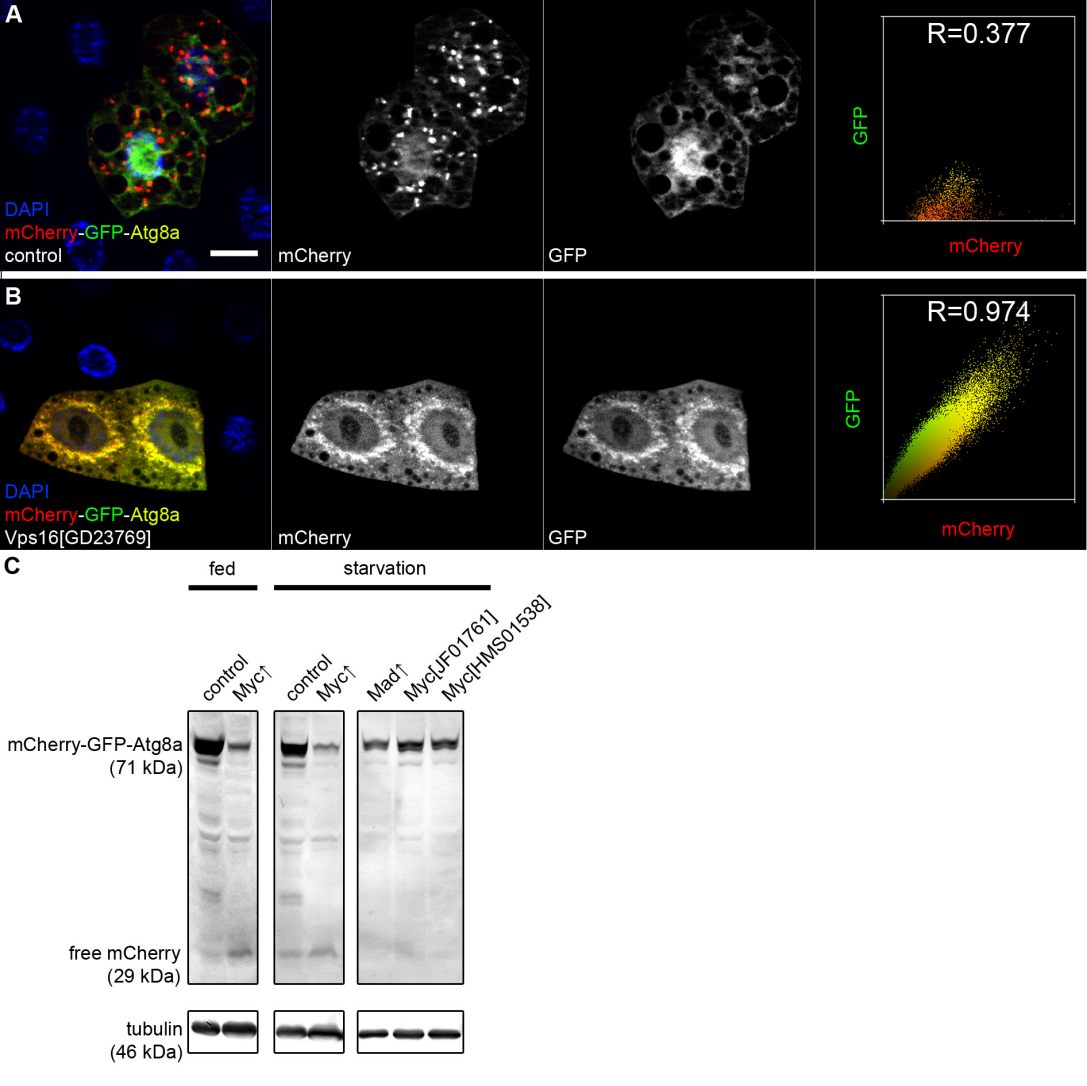
Assays for autophagic flux. (A) and (B) The tandem tagged mCherry-GFP-Atg8a reporter labels autophagosomes as yellow (positive for both GFP and mCherry), while autolysosomes appear mostly red due to faster lysosomal quenching of GFP than mCherry. Knockdown of *Vps16A* (encoding a HOPS tethering complex subunit) prevents autophagosome-lysosome fusion, so all dots are double positive for GFP and mCherry in fat body cells of a starved larva (panel B), compared to the larger dots that are only positive for mCherry in control cells (panel A). Note that this change is also obvious in the pixel intensity correlation profiles calculated from these images (left panels) and in Pearson correlation coefficients (R values). (C) Overexpression of the transcription factor Myc promotes autophagic degradation both under well-fed and starved conditions, as shown here by the increased conversion of mCherry-GFP-Atg8a into free mCherry in larval fat body lysates. Note that the level of the full-length protein is reduced accordingly. Vice versa, inhibition of Myc activity by overexpression of Mad, or by silencing *Myc* using either of two independent RNAi lines, reduces the autophagic degradation-dependent conversion of mCherry-GFP-Atg8a into free mCherry. Bar in panel A equals 20 μm for A, B.

The tandem tagged version of Atg8a is very frequently used for estimating autophagic flux in mammalian cells. This assay is based on the fact that GFP is quenched more rapidly in autolysosomes than mCherry, which made it possible to study autophagic degradation, first in cultured human cells and later in *Drosophila* [15,39]. Phagophores and autophagosomes appear yellow (both green and red) in merged images, whereas autolysosomes are labeled mostly red by this reporter (Fig. 5A and B) [15,18,39,40,63,74]. Thus, this assay is simple and easy to carry out, even though it is not as widely used in *Drosophila* as in cultured mammalian cells. Potential reasons for why it is not so popular in flies may be that it needs to be crossed into the genotype of interest, and that in mosaic analysis, one needs to mark mutant/RNAi clone cells which usually requires the use of GFP or RFP. An alternative solution to this latter problem is to use the flux reporter itself to identify cell clones (although in this case the surrounding tissue is not expressing the reporter, so it cannot be used as a built-in control). Nevertheless, we think that the tandem tagged Atg8a reporter is a very useful tool for estimating autophagic flux in the fly.

The so-called conversion assay is one of the most commonly used methods for studying autophagy in yeast, and recent data shows that it works well in *Drosophila*, too. This technique takes advantage of the fact that the tightly packed, globular structure of fluorescent tags is less accessible for lysosomal proteases compared to the tagged protein (Atg8a, p62, LC3 etc), and thus GFP- or mCherry-tagged reporters are converted into free GFP/mCherry within autolysosomes if autophagy progresses normally. The appearance of the free tag (GFP, mCherry, RFP etc) can then be followed by western blots using an antibody that recognizes the fluorescent tag [8,41,44,75]. Note that the overall level of the uncleaved fusion protein often inversely correlates with that of the free tag in these western blot experiments (Fig. 5C). This method is potentially suitable for following the selective autophagic degradation of other cargoes of interest using specific tagged markers, including ribosomes (ribophagy), mitochondria (mitophagy) and so on [9].

Radioactive isotope labeling of newly synthetized proteins (pulse), and measuring their degradation after a certain amount of time (chase) based on the amount of free radioactive amino acids, has been used in classical studies as a biochemical assay for the average autophagic degradation of long half-life proteins [3]. This technique is based on comparing measurements in the absence and presence of lysosome inhibitors to differentiate between autophagy and other degradation systems. The advent of quantitative proteomic approaches made it possible to employ stable isotope labeling to study the selective autophagic degradation of thousands of individual proteins simultaneously [76]. Recently, the autophagic and autophagy-independent turnover of mitochondial proteins was successfully analyzed by this technique. By feeding adult *Drosophila* with yeast labeled by deuterium-containing leucine, it was possible to measure the rates at which unlabeled proteins were degraded and replaced by labeled ones in various genetic backgrounds [77].

The most sensitive test for quantitatively measuring autophagic flux is the Pho8Delta60 assay in yeast [9]. It is based on a truncated, cytosolic form of the vacuolar/lysosomal enzyme alkaline phosphatase, which is transported to the vacuole by non-specific autophagy, where it is activated by limited proteolysis. Thus, alkaline phosphatase activity measured from cell lysates in a simple enzymatic reaction can be used to establish the rate of bulk autophagic degradation. Unfortunately, a similarly powerful test is not yet available in *Drosophila*.

## 7 Analyzing the role of autophagy, and finding potential new regulators of this process

There are a number of experiments available for studying the role of autophagy beyond estimating autophagic activity/flux. Functional analysis of autophagy is feasible through Atg loss-of-function studies to prove the potential role for this process in a given setting. Null mutants and transgenic RNAi lines are readily available for most Atg genes, and dominant-negative transgenes can also be used for the inhibition of Atg1, Vps34 and Atg4 [21,23,41]. It is generally a good idea to test multiple genes in such experiments, as at least a subset of Atg genes likely function in processes other than autophagy, and not all Atg genes may always be necessary for autophagy in a given cell type [9]. Gain-of-function studies are also feasible, as overexpression of Atg1 has been shown to induce autophagy [23]. Likewise, the brain-specific overexpression of Atg8a was found to promote longevity, probably as a result of counteracting the age-dependent decline in basal autophagy [14]. The importance of selective autophagy in a certain context may also be tested in experiments using p62 mutants or knockdowns, such as in the cases of integrin-dependent hemocyte spreading or during autophagy-mediated protection of retina against expression of the proapoptotic gene *Reaper* [68,75]. However, p62 is also involved in the regulation of multiple signaling routes including Nrf2-mediated antioxidant responses, and not only autophagy but also this latter pathway was recently shown to be required for Myc-induced overgrowth [44].

Drugs modulating autophagy have been traditionally applied to study this process, and some are still often used in cultured cells. These include the previously mentioned bafilomycin to inhibit autophagic degradation, and 3-methyl-adenine to block Vps34 and thus autophagosome formation [9]. Unfortunately, these drugs are toxic and applying them is not feasible in whole animals. Chloroquine is already in clinical use for the (not entirely specific) inhibition of autophagy, and it can be fed to *Drosophila* as described above. Vice versa, pharmacological activation of autophagy by the Tor inhibitor rapamycin was found to improve disease progression by reducing aggregate numbers in Huntington disease models (mice and flies), and to promote longevity [78,79]. The naturally occurring polyamine spermidine appears to increase lifespan in a wide range of organisms, and maintain memory function in *Drosophila* [80,81]. While rapamycin and spermidine influence several processes and are not specific autophagy enhancers, accumulating evidence indicates that autophagy induction is essential for the beneficial effects of these drugs [79,81].

New regulators of autophagy can be identified using numerous different approaches, some of which are based on the autophagy assays described earlier in this paper. *Drosophila* has a long and successful history in the discovery of genes involved in a given process. Genetic screens can be carried out using collections of RNAi, mutant or overexpression lines, and cultured *Drosophila* cells can also be used for systematic knockdown experiments to find hits which can be validated in vivo [19,40,82]. Importantly, the readily available tools for mosaic analysis make it possible to generate and study animals that contain loss-of-function cells for genes whose null mutation causes early lethality during fly development. The biggest advantage of these approaches is that they immediately provide functionally relevant data, although a combination of secondary tests is necessary to establish the role of a given gene product in autophagy. There are a number of widely used methods to provide additional support and mechanistic insight into the potential role of a protein of interest in autophagy, including colocalization and biochemical interaction studies. In other words, if the gene product shows significant colocalization with proteins known to be involved in autophagy and/or they can be coimmunoprecipitated, then such data may indicate that it functions in autophagy. These studies are especially convincing if shown on the level of the endogenous proteins. Proteomics, gene expression and intracellular localization experiments can be also useful for finding new regulators of autophagy, but these tests do not provide functional data. We think that clear loss-of-function (or gain-of-function) phenotypes affecting autophagy on the cellular level are the most important lines of evidence for a given gene’s function in autophagy. It is worth noting that the loss of autophagy is known to also cause certain phenotypes on the organismal level, such as starvation and rapamycin sensitivity, neuromuscular dysfunction based on simple climbing assays, shortening of lifespan, and impaired memory function [6,13,14,40,81,83]. However, these characteristic defects are not necessarily due to a block of autophagy.

## 8 Conclusions and future challenges

The main message of our review is that the proper analysis of autophagy is only possible by using a combination of multiple independent assays. Fortunately, numerous reagents have been developed and tested by the *Drosophila* autophagy community to facilitate such studies in this popular animal model.

Autophagy research has been revolutionized in the past two decades, and the number of related papers published per year is still growing steadily. Much progress has been made in the elucidation of the core mechanisms of autophagy, although we are still far from understanding how Atg proteins and other factors (some of which are likely still unidentified) orchestrate the formation of phagophores and autophagosomes. The concept of selective autophagy started to emerge only 7–8 years ago, with the identification of the first specific cargo receptors. The selective versus bulk nature of autophagy and its role in various physiological and developmental settings is still hardly characterized, and *Drosophila* is an already proven model for important discoveries in these areas.

Atg genes were discovered in yeast using screens for mutations in non-redundant genes that showed very similar loss-of-function phenotypes. The network of proteins involved in the core mechanisms of autophagy expanded considerably with the increasing complexity of metazoans, and a number of Atg gene products turned out to have a role in processes other than autophagy [48,84]. Importantly, several papers indicate that not all Atg genes play an equally critical role in autophagy in certain cell types or developmental contexts [64,85]. There is probably still much room for exploring the autophagy-independent roles of Atg proteins, and alternative autophagy pathways that may not require the full set of established core proteins.

An important question of this field is how autophagy is involved in various human disorders. About 70% of human disease genes have a clear ortholog in *Drosophila*, which makes it possible to model the majority of pathological conditions in flies including aging, cancer, neurodegeneration, myopathy, infection, and so on [5]. Although it will likely take a long time until human patients benefit from *Drosophila* autophagy studies, learning how to cure sick flies may pave the way for new biomedical applications in the long run.
